# The Link between Ankylosing Spondylitis, Crohn's Disease, *Klebsiella*, and Starch Consumption

**DOI:** 10.1155/2013/872632

**Published:** 2013-05-27

**Authors:** Taha Rashid, Clyde Wilson, Alan Ebringer

**Affiliations:** ^1^Analytical Sciences Group, Kings College, 150 Stamford Street, London SE1 9NH, UK; ^2^Department of Pathology and Microbiology, Kings Edward VII Memorial Hospital, 7 Point Finger Road, Paget DV04, Bermuda

## Abstract

Both ankylosing spondylitis (AS) and Crohn's disease (CD) are chronic and potentially disabling interrelated conditions, which have been included under the group of spondyloarthropathies. The results of a large number of studies support the idea that an enteropathic pathogen, *Klebsiella pneumoniae*, is the most likely triggering factor involved in the initiation and development of these diseases. Increased starch consumptions by genetically susceptible individuals such as those possessing HLA-B27 allelotypes could trigger the disease in both AS and CD by enhancing the growth and perpetuation of the *Klebsiella* microbes in the bowel. Exposure to increased levels of these microbes will lead to the production of elevated levels of anti-*Klebsiella* antibodies as well as autoantibodies against cross-reactive self-antigens with resultant pathological lesions in the bowel and joints. Hence, a decrease of starch-containing products in the daily dietary intake could have a beneficial therapeutic effect on the disease especially when used in conjunction with the currently available medical therapies in the treatment of patients with AS and CD.

## 1. Introduction

Ankylosing spondylitis (AS) is regarded as the prototype of seronegative spondyloarthropathies (SpAs) that comprise a group of spondylitis-associated conditions. Other disease entities of SpA include reactive arthritis, psoriatic arthritis, undifferentiated SpA, and arthritis associated with inflammatory bowel disease (IBD), which includes Crohn's disease (CD) and ulcerative colitis (UC) [1]. SpAs are interrelated conditions which share certain associated clinical, laboratory, radiological, and genetic manifestations such as inflammatory back pain caused by spondylitis/sacroiliitis, as well as asymmetric oligoarthritis, enthesopathy, anterior uveitis, positive family history, and association with HLA-B27 genes, but without positivity for the rheumatoid factors.

Although patients with CD usually present with clinical features of bowel involvement, the characteristic presentation in those with AS and spondylitis-associated CD is progressive inflammatory backache with or without other SpA-associated features [2]. 

Both AS and CD affect early age groups and have a world-wide distribution. There are at least one million individuals in the United Kingdom who suffer from some features of AS. The negative impact of AS on the employment [3] and the psychological [4] status of patients with this disease has been well established. The disease in CD can also have an impact on the social status and work abilities of patients, especially in women [5]. Because of these negative impacts on the general health and welfare status of patients with AS and CD, with certain drawbacks of the currently used medical treatments, a search for the causative factor and an alternative therapeutic measure involving eradication of the cause could be helpful in the management of patients with these diseases.

## 2. Genetic Background of AS and CD

A positive family history is one of the key points in defining the characteristics of patients with SpA. In a family study of AS probands and healthy controls in an Icelandic population, it has been shown that there is evidence which might support the existence of common genetic components for AS and IBD. The study demonstrated a risk ratio of 3.0 and 2.1 in the first and second-degree relatives, respectively, for the occurrence of AS in families of probands with IBD, and with the occurrence of IBD in families of patients with AS [6]. In a more recent study, it has been shown that there is genetic overlap across the autoimmune diseases including also AS and IBD [7]. It appears, therefore, that certain common genetic factors might act in the development of both diseases in AS and CD.

The frequency of association of HLA-B27 allelotypes in patients with AS is considered as the strongest genetic link with any disease which have been encountered in the field of rheumatology [8]. This genetic bond was discovered in the early 1970s, where more than 95% of patients with AS have been found to possess HLA-B27, whilst the frequency of this gene in the general population was below 10% [9, 10]. Other diseases in the SpA group have lower but different degrees of associations with this allelotype. For example, the frequency of this allelotype in patients with IBD/CD without associated arthritis is comparable to those of the normal population but increases to 40%–60% in those patients with spondylitis/sacroiliitis [11]. These data show that a spondyloarthropathic patient presenting with spinal involvement has a higher chance of possessing HLA-B27 genes than those presenting with peripheral joints involvement only. Apart from HLA-B27, other genes, whether located within or outside the major histocompatibility complex region, have also been implicated in the aetiopathogenesis of both AS [12] and CD [13]. 

## 3. The Link between AS and CD

There are certain characteristics linking AS and spondylitis-associated CD together based on sharing some of the genetic, clinical, immunological, and microbial features [14]. Furthermore, most if not all SpA conditions are thought to have a fundamental link with the gut lesions and enterobacterial microbes [15]. For example, around 10 percent of patients with AS have overt IBD, whilst 70% of AS patients have subclinical terminal ileitis [16]. Axial and peripheral arthritis can occur in up to 30% of patients with CD [17], and the prevalence of AS might increase to up to 6% in patients having CD [18]. Moreover, HLA-B27 positive patients with IBD were shown to have higher chance of developing AS compared to those without IBD [19]. Rats transgenic for HLA-B27 spontaneously develop a chronic inflammatory disease that resembles, both clinically and histologically, the human SpA, while control rats transgenic for HLA-A2 do not develop such an illness [20]. These results support the role of gut and intestinal flora in the development of SpAs mainly in genetically susceptible individuals such as those possessing the HLA-B27 genes. Nevertheless, up to 30 percent of patients with unclassified HLA-B27-positive inflammatory rheumatic diseases develop into one form of definite SpA group such as AS, IBD, or reactive arthritis [21]. It has also been reported that more than half of patients with undifferentiated SpA will develop AS over certain period of time [22].

It would appear from these results that both HLA-B27 and gut inflammation play a pivotal role in the development of SpAs, especially AS and CD, and that the main aetiopathogenetic process is triggered by genetic and environmental (mainly microbial) factors.

## 4. Evidence of Subclinical Microbial Infections in AS and CD

The first evidence of the epidemiological link between infection and SpA was detected in the early twentieth century where a triad of symptoms consisting of urethritis, conjunctivitis, and arthritis, being termed as Reiter's syndrome, was detected in a group of soldiers living under unhygienic condition during the First World War following several bouts of infections [23]. This condition, however, was later recognized as a form of reactive arthritis, which is known to be preceded by infections with enterogenic or urogenital bacteria [24]. 

Previous review analyses have shown that the results of molecular, immunological, and microbiological studies could establish the link between subclinical *Klebsiella* infections and the aetiopathogenesis of both AS [25, 26] and CD [14, 27]. Evidence for these links is summarized as follows.

### 4.1. *Klebsiella* and AS

- Rabbits immunised with HLA-B27-positive lymphocytes showed increased haemagglutinating activity against sheep red cells coated with *Klebsiella* lipolysaccharide and these elevations were statistically significant when compared to serum samples obtained from the same rabbits before immunisation [28]. It has also been shown that HLA-B27 positive allogeneic human tissue typing sera were binding more significantly to *Klebsiella* microbes in comparison to sera containing other HLA-tissue specific antibodies [29].
- Anti-HLA-B27 monoclonal antibodies bind to *Klebsiella*, *Shigella*, and *Yersinia*, enterobacterial agents [30], indicating the existence of some shared or cross-reactive antigens among these microbes. Other anti-B27 monoclonal antibodies were found to bind more preferentially to *Klebsiella* than to *Shigella* and *Yersinia* microbial antigens [31].
- A homologous amino acid sequence, QTDRED, present in HLA-B27 was found to have molecular similarity to another sequence within *Klebsiella pneumonia* nitrogenase reductase enzyme [32]. More homologous amino acid sequences were found to exist between *Klebsiella* secretion products and self-antigens. A quadrimeric DRDE sequence present in *Klebsiella* pullulanase pul-D enzymes shares homology with a DRED sequence present in HLA-B27 molecules. Another homologous sequence was found to exist between *Klebsiella* pullulanase pul-A enzymes, which have the tripeptide “Gly-X-Pro” sequence, and the same antigen is present in collagen types I, III, and IV [33].
- Various immunological studies carried out by independent groups from 16 different countries have shown that antibodies against *K. pneumonia* and/or cross-reactive self-antigens but not against other microorganisms are significantly elevated among patients with AS when compared to patients with other diseases or to healthy individuals [34].
- Levels of anti-*Klebsiella* antibodies were found to be significantly higher in the serum than in the synovial fluid samples taken from AS patients [35]. The sources of these antibodies are from extra-articular regions such as the lymph nodes draining the gut [36].
- Serum samples taken from active AS patients were found to possess significant in vitro cytotoxic activities when compared to sera taken from patients with RA or healthy controls. Increased percentage of lysis is present in sheep red blood cells which have been coated with *Klebsiella* cross-reactive antigens such as HLA-B27 synthetic peptides, QTDRED [37].
- Antibodies to *Klebsiella* nitrogenase reductase peptides, QTDRED, were shown to bind preferentially to the synovial tissues of AS patients when compared to those from patients with other rheumatic diseases [38].
- *Klebsiella* bacteria have been isolated by different independent groups more significantly from the bowel of active AS patients when compared to controls [39–42]. These findings, however, were not confirmed by other groups [43, 44]. The discrepancies in these results could be explained by the differences in the methods of collections and cultures of the faecal specimens and the disease activity status. Furthermore, in a study by a group from Finland it was shown that elevated levels of IgA anti-*Klebsiella* antibodies in patients with AS correlated with the degree of gut inflammation [45].

It is well documented that there is a strong link between gut inflammation [46] and/or AS [47, 48]. The level of total [49] and secretory IgA immunoglobulins [50] increased in the majority of patients with AS. Moreover, there is evidence for elevated levels of IgA, particularly secretory IgA antibody against *Klebsiella* antigens [51–53] or *Klebsiella* cross-reactive antigens [54] in active patients with AS. The results of these studies linking *Klebsiella*, collagen, and HLA-B27 to AS could explain some of the predominant characteristic clinical, genetic, and immunological features present in the patients with this disease (Table 1).

### 4.2. *Klebsiella* and CD

**Figure 1:**
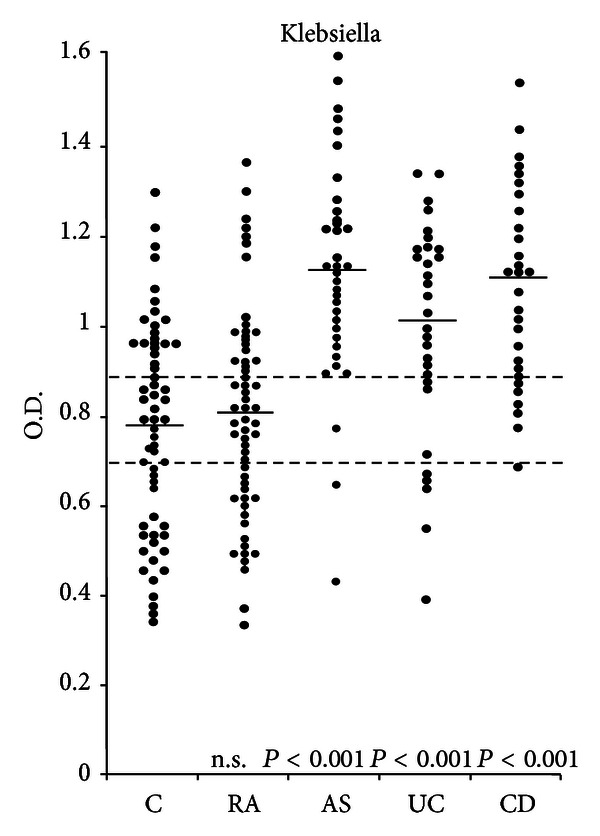
Total immunoglobulin (IgM, IgA, IgG) levels against *Klebsiella pneumonia* in healthy controls (C) and in patients with rheumatoid arthritis (RA), ankylosing spondylitis (AS), ulcerative colitis (UC), and Crohn's disease (CD) (with permission).

- Previous studies showed that *Klebsiella* microbes have been isolated from the large bowel specimens in more than 25% of patients with CD [55], and that relapses of the disease in patients with CD were found to be associated with *Klebsiella* colitis [56].
- Serological and immunological studies on the link between *Klebsiella* and patients with IBD/CD were carried out by various groups from six different gastroenterology centres. (a) Elevated levels of anti-*Klebsiella* and anti-*Yersinia* antibodies were observed in patients with CD and UC from Birmingham when compared to corresponding healthy control subjects [57]. (b) Both groups of patients with IBD and AS from Glasgow [58] and Edinburgh [59] were shown to have significantly elevated levels of anti-*Klebsiella* IgA antibodies when compared with corresponding healthy controls. (c) Three consecutive studies from London and Winchester in the UK have shown similar results. In one study, anti-*Klebsiella* antibody levels were found to be significantly elevated in patients with AS, CD, and UC when compared to healthy or diseases controls, whilst no such elevation in antibodies was observed against *Escherichia* or anaerobic intestinal microbes (Figure 1) [60]. In a second study, elevated levels of class-specific antibodies against many capsular serotypes of *Klebsiella* bacteria have been observed in patients with CD and AS when compared to patients with coeliac disease or to healthy control subjects [61]. In the third study, class-specific antibodies against *Klebsiella* microbes and cross-reactive collagen types I, III, IV and V were found to be significantly elevated in patients with AS, as well as in early and advanced cases with CD when compared to healthy subjects. In the same study, serum samples from CD patients had shown a positive correlation between antibody levels to *Klebsiella* and types I, III, and IV of collagen [62].

Furthermore, experimental studies from Nagakute in Japan have shown that collagen-induced enterocolitis [63] and arthritis [64] were both observed in animals when immunised with homologous colonic extracts and collagens together with *Klebsiella* lipopolysaccharides.

## 5. Aetiopathogenic Mechanism Linking *Klebsiella* to AS and CD

Molecular mimicry or cross-reactivity hypothesis is suggested to be the main mechanism that can link *Klebsiella* with the initiation and development of AS and spondylitis-associated CD [65]. Evidence obtained from other diseases such as rheumatic fever [66] and primary biliary cirrhosis [67] indicates that molecular mimicry is more than an epiphenomenon, whereby humoral and/or cellular immune responses are consistently detected against targeted tissues at the pathological sites in patients with these conditions. 

The types of cross-reactive antibodies produced following *Klebsiella* infections will determine the anatomical location of the pathological lesions, especially in AS. Some antibodies are reacting with HLA-B27, an antigen which is expressed in most articular tissues inside the synovial joints, whilst other antibodies are reacting with types I, III, and IV of collagen, which form an important component of the spinal tissues where the pathological lesions are located. The binding of these *Klebsiella* cross-reactive antibodies, when present in high titres, triggers inflammatory cascades such as the complement system together with the production of various cytokines resulting in the pathological changes with consequent fibrosis, calcification, and new bone formation leading to the development of classical AS. Moreover, the raised level of HLA-B27 antigen expressions on the targeted tissues in patients with AS [68] will make these molecules more accessible and hence will increase the chance of their binding to anti-*Klebsiella* cross-reactive antibodies. 

## 6. Starch and Gut Microbes

The main substrate that is necessary for the growth of colonic microbial agents includes starch and complex carbohydrates which are usually available in considerable amounts in the bowel. In a study, carried out by a group from Minnesota, using hydrogen breath tests as an index of carbohydrate absorption in healthy individuals, up to 20% of a test meal of starch was found to be available for metabolism by the colonic microflora [69]. It has been found also that up to 10%, of consumed starch can escape the absorption in the small bowel [70], indicating that a considerable proportion of dietary starch reaches the large intestine. In another experimental study it has been shown that a significant increase in the total bacterial population of enterobacterial microbial agents was noticed in the faeces of rats which have been fed diets containing resistant potato starch when compared to those taking rapidly digestible waxy maize starch [71]. 

## 7. *Klebsiella* and Starch-Debranching Enzymes

Starch or macromolecular polysaccharides must initially be hydrolyzed to smaller substrates in order to be transported into cells. To accomplish this hydrolytic and transportation process, bacteria usually use their carbohydrate-degrading enzymes, such as pullulanases and isoamylases [72]. 

**Figure 2:**
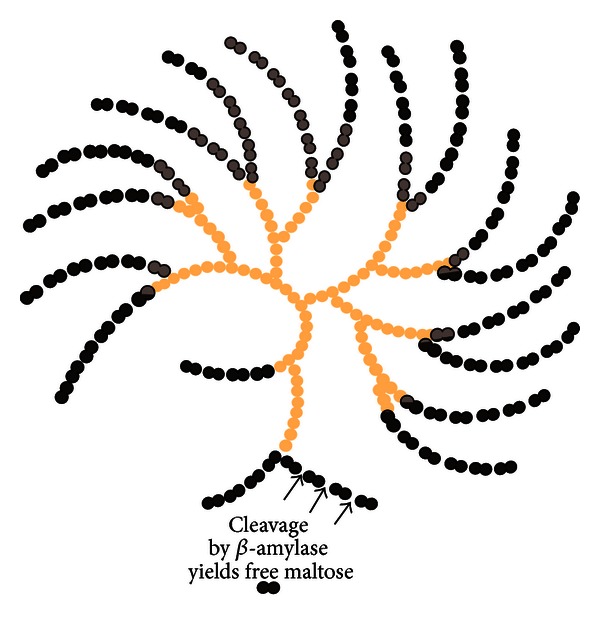
Structure of amylopectin showing a branched carbohydrate polymer and the site of *β*-amylase action yielding free maltose molecules (with permission).

**Figure 3:**
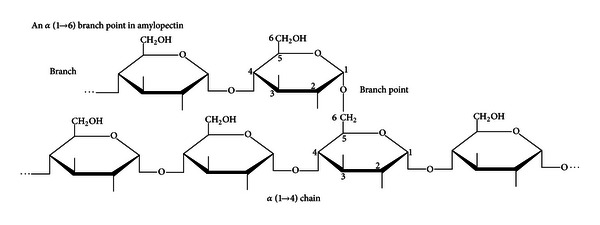
Amylopectin chemical structure showing the point of action by *Klebsiella* pullulanase enzyme on the *α*-(1→6) links (with permission).

The starch molecules, which consist of approximately 20% amylose and 80% amylopectin glucose polymers (Figure 2), are catalyzed by amylases, cyclodextrinases, glucosidases, and other starch debranching enzymes such as bacterial pullulanases [73]. Amylose is a linear polymer consisting of *α*-(1→4) links between glucose residues and these can be readily hydrolysed by amylases present in digestive enzymes. Amylopectin, however, is a branched polymer consisting of linear sequences of amylose like chains linked by *α*-(1→6) side chain giving rise to a branched structure (Figure 3), which can be broken down by *Klebsiella* pullulanase but not by digestive enzymes. Hence, the digestion of starch in the small bowel is limited by inability of luminal digestive enzymes in the gut to break *α*-(1→6) bonds of amylopectin and thereby giving rise to formation of “hard starch” which accumulates in the colon.

*Klebsiella *can survive in harsh environments exploiting some of its enzymatic degrading products, which are required for the protection, maintenance and survival of these microbes. Apart from other enzymatic products such as nitrogenase reductase, *Klebsiella* can also produce starch-hydrolysing and debranching pullulanase enzymes. *Klebsiella* can utilize starch as the sole carbon and energy source via two metabolic routes. The first one involves the extracellular degradation into linear maltodextrins by hydrolysis of the glycosidic bonds via the cell surface-associated pullulanase and then the subsequent cleavage of the glycosidic linkages by the action of the extracellular glycosyltransferase [74]. A fraction of the total dietary starch consumed daily in humans resists digestion by pancreatic amylase in the small intestine, thereby, reaching the colon [75]. This form of undigested or resistant starch is usually fermented by human gut microflora, providing a source of energy and carbon for more than 400 species of bacteria present in colon [76]. 

A group from Los Angeles had shown that the mean number of faecal *Klebsiella* concentrations in individuals taking high carbohydrate/low protein diet was forty times higher than in those having low carbohydrate/high protein diet [77]. Similarly, the mean number of *Klebsiella* was found to be ten times higher when incubated with simple carbohydrate products such as sucrose, lactose, and glucose than with eleven different amino acids [78]. These results indicate that complex carbohydrates such as starch-containing products are necessary for the growth, replication, and persistence of many enterobacterial agents including *Klebsiella* microbes in the large bowel. 

## 8. Potential for the Use of Low Starch Diet in AS and CD Patients

The current medical therapeutic agents used in patients with AS [79] and CD [80] include nonsteroidal anti-inflammatory and immunosuppressive drugs, as well as biological agents. These treatments, however, cannot reverse the existing destructive spinal lesions and might be associated with deleterious side effects [81, 82]. Therefore, implementation of other therapeutic measures especially those involving the means for effective eradication of the causative agents by using a low starch diet intake and possibly antibiotics together with the currently used medical treatments could have a beneficial effect in the management of patients with AS and CD.

These data support the causative effect of high starch consumption and the beneficial effect of low starch intake in patients with SpAs, especially those with AS or IBD. For example, in a previous study on a group of UC patients, analyses of the contents of surgically removed ileocaecal regions have shown that the ileostomy fluid contained significant amount of monosaccharides and disaccharides [83]. These simple carbohydrate products detected in the ileostomy fluid would appear to be derived from starch. In another prospective longitudinal study, the influence of dietary factors was examined in a group of Italian patients with IBD and a group of healthy controls well matched for age, sex, and location of living. The results showed that patients with CD and UC have an increased consumption of the total carbohydrate and starch with a significantly higher relative risk compared to healthy individuals [84]. In a later review analysis of the literatures on the daily intake of diets and their relation to intestinal microbial flora in patients with IBD, it was shown that a considerably large amount of data show an association between increased intake of westernized carbohydrate food, high intestinal microbial load, and the occurrence of IBD [85].

In a longitudinal open study carried out in a group of 36 patients with active AS in “London AS Clinic,” most of the patients had shown reductions in their erythrocyte sedimentation rates and total IgA concentrations, as well as a drop in their intake for the anti-inflammatory medicines after a nine-month followup following a decrease the dietary intake of starch [86]. It appears that in both IBD and AS, an interaction between the gut microflora and the mucosa is a possible contributor to the development of these diseases. These data results support the notion that an increase in the bulk of potentially pathogenic organisms such as *Klebsiella* in the faecal microflora due to high starch consumption could help in the initiation and development of both AS and CD. It seems, therefore, that an exclusion of a diet containing complex carbohydrates such as starch, but not simple carbohydrate-containing foods such as glucose or sucrose, might inhibit the growth of *Klebsiella* and could ameliorate the disease process and activity in patients with AS and CD.

## 9. Conclusions

AS and CD are shown to be two interrelated conditions mainly based on the existing genetic and immunological features. The main pathogenetic mechanism which can explain this linkage is “molecular mimicry” or “cross-reactivity” between *Klebsiella pneumonia* and target tissues. It appears that starch is the main source of *Klebsiella* growth in the colon. Hence, increased consumption of starch-containing foods by genetically susceptible individuals such as those possessing HLA-B27 genes could result in the initiation and development of AS or spondylitis-associated CD. Dietary manipulation in the form of low starch diet intake can be included in the management of patients with AS or CD, especially when used in conjunction with the current medical therapeutic measures.

## Figures and Tables

**Table 1:** Possible explanations for some of the predominantly associated features in “AS.”

“AS” associated features	Suggested explanation
Fluctuation in the course of the disease and low concordance rates in identical twins	Involvement of nongenetic, environmental, factors in the disease pathogenesis

High association with HLA-B27	Cross-reactivity of these antigens with *Klebsiella *

Predilection for involvement of the sacroiliac and vertebral joints	Lymphatic drainage plexus of the bowel (containing *Klebsiella* antibodies) is in close proximity to the sacro-iliac joints

Polyarticular joint involvement	Cross-reactivity between collagen I, III, and IV fibres and *Klebsiella *

Associated uveitis	Cross-reactivity of uveal tract tissues with *Klebsiella *

Associated enthesitis	A site of inflammation in the early cases of AS

Increased total IgA and secretory IgA in sera of AS patients	Enhanced mucosal immune response due to subclinical bowel infections by *Klebsiella *

Higher onset of AS among young people	Increased intake of starch diet among young age groups
